# N-Acetylaspartate Drives Oligodendroglial Differentiation via Histone Deacetylase Activation

**DOI:** 10.3390/cells12141861

**Published:** 2023-07-14

**Authors:** Alessandra Dominicis, Alice Del Giovane, Matteo Torreggiani, Antonella Damiana Recchia, Fabio Ciccarone, Maria Rosa Ciriolo, Antonella Ragnini-Wilson

**Affiliations:** 1 Department of Biology, University of Rome Tor Vergata, 00133 Rome, Italyalice.delgiovane.adg@gmail.com (A.D.G.);; 2 IRCCS San Raffaele, 00166 Rome, Italy

**Keywords:** remyelination, epigenetics, histone deacetylases (HDACs), multiple sclerosis (MS), N-acetylaspartate (NAA), neurodegeneration, Oli-neuM

## Abstract

An unmet clinical goal in demyelinating pathologies is to restore the myelin sheath prior to neural degeneration. N-acetylaspartate (NAA) is an acetylated derivative form of aspartate, abundant in the healthy brain but severely reduced during traumatic brain injury and in patients with neurodegenerative pathologies. How extracellular NAA variations impact the remyelination process and, thereby, the ability of oligodendrocytes to remyelinate axons remains unexplored. Here, we evaluated the remyelination properties of the oligodendroglial (OL) mouse cell line Oli-neuM under different concentrations of NAA using a combination of biochemical, qPCR, immunofluorescence assays, and in vitro engagement tests, at NAA doses compatible with those observed in healthy brains and during brain injury. We observed that oligodendroglia cells respond to decreasing levels of NAA by stimulating differentiation and promoting gene expression of myelin proteins in a temporally regulated manner. Low doses of NAA potently stimulate Oli-neuM to engage with synthetic axons. Furthermore, we show a concentration-dependent expression of specific histone deacetylases essential for MBP gene expression under NAA or Clobetasol treatment. These data are consistent with the idea that oligodendrocytes respond to lowering the NAA concentration by activating the remyelination process via deacetylase activation.

## 1. Introduction

Damage to myelin sheaths results in impaired axonal saltatory nerve conduction and neurodegeneration. Demyelination of axons is the major cause of functional loss in the adult brain, and it can occur because of chronic autoimmune diseases such as multiple sclerosis (MS) or because of hypoxia, inflammation, dementia, or traumatic brain injury (TBI). N-acetylaspartate (NAA) is one of the most abundant amino acid derivatives present in the adult CNS, rising to about 10 mM in gray matter-rich regions. Brain NAA is mainly present in immature oligodendrocytes [1,2,3]. Brain NAA is synthesized in neurons and catabolized into aspartate and acetate by aspartoacylase (ASPA) in oligodendrocytes (OLs). NAA physiological functions in glial cells have been evaluated for their role in myelin, as NAA provides acetyl groups for lipid synthesis in myelinating rat brains in vivo [4,5,6]. Despite its abundance, NAA can drop by over 20% within 1 min during severe TBI, and average levels were 40% lower than those measured in healthy subjects 70 h post-injury. It has been established that the average concentration of NAA is in the range of 10 to 20 μM in extracellular fluid obtained from both TBI-injured and uninjured subjects, suggesting a substantial NAA gradient from the intracellular to the extracellular environment [7]. Demyelination and axonal degeneration are hallmarks of multiple sclerosis (MS). Secondary Progressive MS patients (SPMS), but not Relapsing-Remitting MS patients (RRMS), are characterized by low NAA levels. Reduced NAA levels in the normal-appearing white matter in patients with MS are a feature of progression [8]. In healthy individuals, the remyelination of damaged myelin occurs spontaneously. Glial cells (microglia, astrocytes, and oligodendrocytes) are master regulators of the remyelination process in vitro and in vivo in response to neuronal damage [9,10]. Parenchymal oligodendrocyte progenitor cells (pOPC), expressing PDGFRα and NG2 proteoglycan, generate myelinating oligodendrocytes (OLs) that contribute to remyelination following demyelinating insults [11,12]. How changes in NAA concentration in the brain affect pOPC metabolism and their ability to regenerate damaged myelin remains unclear. Here, we have explored the idea that low NAA concentrations might influence oligodendroglia regenerative capabilities.

Remyelination is a temporally regulated program. Genetic fate-mapping and single-cell analyses show that pOPC generate myelinating OLs that contribute to remyelination forming a thin myelin sheath around demyelinated axons [12]. In addition, neural stem cells (NSCs) of the subventricular zone (SVZ) participate in the remyelination process by proliferating, migrating, and differentiating into oligodendrocyte precursor cells (OPCs) [13,14,15,16]. Recently, it has been shown that pOPC could act as primary responders to demyelination, while OPCs derived from NPCs might contribute to repopulating the OPCs pool following their depletion [17] and OPCs derived from NSC compete at the remyelination site [18]. Remarkably, the g-ratio of remyelinated axons by NPC-derived OPCs is significantly thicker than that generated by pOPCs [13,19]**.** The extracellular stimuli that promote oligodendroglia reactivation at the site of remyelination remains unclear [20,21,22].

Overall, pharmacological therapies for chronic demyelinating diseases such as MS also need to be evaluated for their chronic use. Therefore, the reduction in time and dose and the choice of the moment of drug administration are additional goals to be achieved in remyelination therapies [10,20,21]. Metabolomics studies identified metabolites that increase during OPC differentiation, with the idea that naturally occurring metabolites can boost adult brain remyelination. From these studies, taurine was selected as an abundant natural metabolite potentially able to boost remyelination in combinatorial therapy in RRMS [23]. Following this line of research, with the aim of identifying metabolites that decrease under pathological conditions and that can be potentially supplemented in combination with remyelination agents in co-therapies, we recently focused our attention on an abundant brain metabolite NAA.

It is unclear what percentage of acetyl-CoA derived from NAA is associated with myelin repair and remyelination after brain injury [1,5,8]. Providing immediate treatment to TBI victims can help prevent the development of secondary brain damage due to severe inflammation, edema, and delayed cell death [1,5]. SPMS patients with features of disease progression, but normal-appearing white matter, show decreased NAA levels. These changes are accompanied by myelin decompaction, a typical feature in SPMS patients [8]. Impairment of NAA catabolism in oligodendrocytes consequent to a genetically inherited ASPA deficiency (Canavan disease) leads to leukodystrophy and neurodegeneration [24,25]. Altogether, these data suggest a strict correlation between NAA and the myelination process. However, how changes in NAA levels or its derivatives play a role in regulating the remyelination processes remains largely unanswered [24,26].

Here, we have used the oligodendroglial mouse cell line Oli-neuM to evaluate several parameters indicative of the ability of OLs to remyelinate axons under stimulation. The Oli-neuM cell line, unlike other oligodendroglial cell lines [27], express myelin genes in a timely regulated manner and differentiate in OL capable to engage synthetic axons. Oli-neuM has been previously used successfully for phenotypical screens aimed at identifying promyelinating drugs [28] with regenerative properties in vivo [10]. Here, we tested the promyelinating abilities of NAA at concentrations from 1 µM to 2 mM using immunofluorescence microscopy (IF), quantitative PCR (qPCR), and biochemical tools. Altogether, our data show that a reduction in the NAA levels from 2 mM to 20 µM promotes oligodendroglia differentiation, enhances remyelination, and promotes protein deacetylation via histone deacetylase (HDAC) gene expression. Moreover, we show that inhibition of HDACs activity impairs preOL maturation. All together our data suggests that NAA acts as a signaling molecule for pOPC maturation in OLs and low NAA dosages in their extracellular space can induce the remyelination process.

## 2. Materials and Methods

### 2.1. Cell Culture and Media

The Oli-neuM line (Cellosaurus ExPASy CVCL_VL76) was grown in either growth (GM) or differentiation medium (DM) containing 500 µg/mL geneticin (G418, Gibco^TM^, Thermo Fisher Scientific, Waltham, MA, USA, 10131027) and maintained at 37 °C in 5% CO_2_, as previously described [28,29,30].

### 2.2. Compound Treatments

N-Acetyl-L-aspartic acid (Sigma-Aldrich, Burlington, MA, USA) was dissolved in water containing 8% NaOH and used at a final concentration indicated in the text. Clobetasol (Prestw-781) and Gefitinib (Prestw-1270) were purchased from Prestwick Chemical Library^®^, Illkirch-Graffenstaden, France and used at a final concentration of 10 µM or 1 µM, respectively, as previously described [30]. The HDAC inhibitor ACY-738 (HY-19327, MedChemExpress, Monmouth Junction, NJ, USA) was dissolved in 100% DMSO at a final concentration of 10 mM and used at a concentration of 10 µM, according to [31]. After which, 0.5% DMSO max was added to NAA or vehicle treatments in all experiments, while Clobetasol, Gefitinib, or ACY-738 were used in parallel experiments as controls. Drug treatments were administrated in DM for 48 h to cells left to grow in GM for 24 h. Culturing and time of drug treatments have been established previously based on the timing and concentration for optimal MBP expression in Oli-neuM [29,30]. Engagement tests were performed as previously described in growth chambers containing polystyrene (PS) microfibers of 2–4 µm. For microfiber engagement analysis, the cells were treated for a minimum of 72 h, according to previously established protocols [28,29].

### 2.3. Quantitative Immunofluorescence Microscopy Analysis

IF microscopy was carried out in 96-well plates (Grainer bio-one, Kremsmünster, Austria, 655090 CHIMNEY WELL, µCLEAR^®^, NERO, CELLSTAR^®^, TC). Cells were fixed and processed for IF, as previously described [28,29]. Acquisition was performed at 20× magnification (HCX PL FLUOTAR 20 × NA 0.4) using a Leica DMI6000 B epifluorescence inverted microscope (Leica Microsystems, Wetzlar, Germany), equipped with Leica Application Suite X and Matrix Screener software (version 3.0) for automated image acquisition. Micrographs were analyzed for quantification and statistical analyses with ScanR Analysis software (version 1.1.0.6 or 3.0; Olympus, Tokyo, Japan), as previously described [28,29,30]. Hoechst 33342 (H3570, Thermo Fisher Scientific Inc., Waltham, MA, USA) and phalloidin (A12380; 1:40; Thermo Fisher ScientificInc.,, Waltham, MA, USA) staining were performed to detect nuclei and actin cytoskeleton. Rat anti-MBP (MCA409S, 1:100; AbD Serotec, Hercules, CA, USA), Rabbit anti-Ki67 (NB110-89717SS, 1:200; Novus biologicals, Minneapolis, MN, USA), and mouse anti-Ac-lysine (AKL5C1 sc-32268, 1:300, Santa Cruz Biotechnology, Inc., Dallas, TX, USA) were used as primary antibodies (Abs) and Alexa Fluor 488 or Alexa Fluor 546 as secondary Abs (Thermo Fisher Scientific Inc., Waltham, MA, USA), as indicated in the text. Image analysis was performed as previously described [30]. Membrane enlargement was determined using the Max Feret parameter of ScanR analyses software (Olympus, Soft imaging solution Gmbh DE, v. 2.1 and 3.0) as we previously described [28].

### 2.4. Evaluation of Cell Engagement in PS Microfibers

Cell culture chambers containing electro spun polystyrene microfibers were prepared as indicated in [29], UV-sterilized before use, and pre-treated with 10 µg/mL fibronectin (F0895; Sigma-Aldrich, Burlington, MA, USA). A total of 60,000 Oli-neuM cells were seeded in growth medium. After 24 h, the medium was exchanged for either DM supplemented with 0.5% DMSO (vehicle), 20 µM NAA, or 10 µM Clobetasol (as positive control). After 72 h at 37 °C in 5% CO_2_, cells were processed for fixation and IF microscopy analyses. MBP levels were determined using the anti-MBP Ab as indicated above and in the text. Acquisition and engagement analyses were performed as described in [28,29].

### 2.5. Total RNA Extraction and qPCR

Typically, for gene expression studies, 125,000 Oli-neuM cells/well were seeded in GM in 12-well plates. After 48 h, GM was substituted with DM, to which treatment or vehicle (DMSO 0.5% max) was added as described in the text. After 48 h, RNA extraction, quantification, and cDNA production were performed using RNA-Solv Reagent (R6830-01; VWR, Radnor, PA, USA), according to the manufacturer’s instructions. Typically, 2 µg of the RNA per sample was retro-transcribed using the High-Capacity cDNA Reverse Transcription Kit (4368814; Thermo Fisher Scientific Inc., Waltham, MA, USA), as previously described. qPCR was performed using SYBR Green Technology and the QuantStudio R 3 Real-Time PCR System (Applied Biosystems R, Thermo Fisher Scientific Inc., Waltham, MA, USA). Primer pairs used with StoS Quantitative Master Mix 2X SYBR Green-ROX (GeneSpin Srl, Milan, Italy) are indicated in Table 1. GAPDH was used as an endogenous control to normalize data. Typically, 50 ng of cDNA per sample was used per reaction. qPCR was performed in triplicate in MicroAmp Fast Optical 96-Well Reaction Plate (Applied Biosystems R, Waltham, MA, USA). The 2^−ΔΔCT^ relative quantification method was used to determine fold change in expression. This was done by normalizing the resulting threshold cycle (CT) values of the target mRNAs to the CT values of the endogenous control GAPDH in the same samples (ΔCT = CT target–CT GAPDH) and by further normalizing to the control (ΔΔCT = ΔCT–ΔCT vehicle). The fold change in expression was then obtained (2^−ΔΔCT^), and log_2_ of the fold change is represented in the plots.

### 2.6. Crude Extract Preparation and Immunoblot Analysis

Typically, 2.75 × 10^5^ Oli-neuM cells were seeded in 6-well plates in GM media, and cells were grown to 70% confluence. Treatments were performed for 48 h in DM. For immunoblot analyses, the following antibodies diluted in TBS and 4% BSA were used: anti-actin (66009-1-Ig, 1:5000; Proteintech Group Inc., Rosemont, IL, USA); AbD Serotec (Bio-Rad Laboratories, Hercules, CA, USA): anti-MBP (MCA409S, 1:200). Cell extract preparation and immunoblot analyses were performed as previously described [28,29].

### 2.7. Statistical Methods

In studies performed in 96-multiwell plates (immunofluorescence and qPCR), three experimental replicates per sample were spotted in each plate, and the mean values were considered as one biological replicate. The mean values ± SEM obtained from at least three biological replicates were considered for statistical analyses using GraphPad Prism (San Diego, CA, USA) tools. Effects of each drug treatment versus its internal control (vehicle) in immunofluorescence experiments and qPCR data were analyzed by paired two-tailed Student’s *t*-test, while one-way analysis of variance (ANOVA) with Tukey’s tests (as indicated in figure legends) was performed to determine statistically significant differences among multiple single or combined treatments.

## 3. Results

### 3.1. Oligodendroglia Differentiation and MBP Expression Are Stimulated by 200 mM NAA Treatment

**Figure 1:**
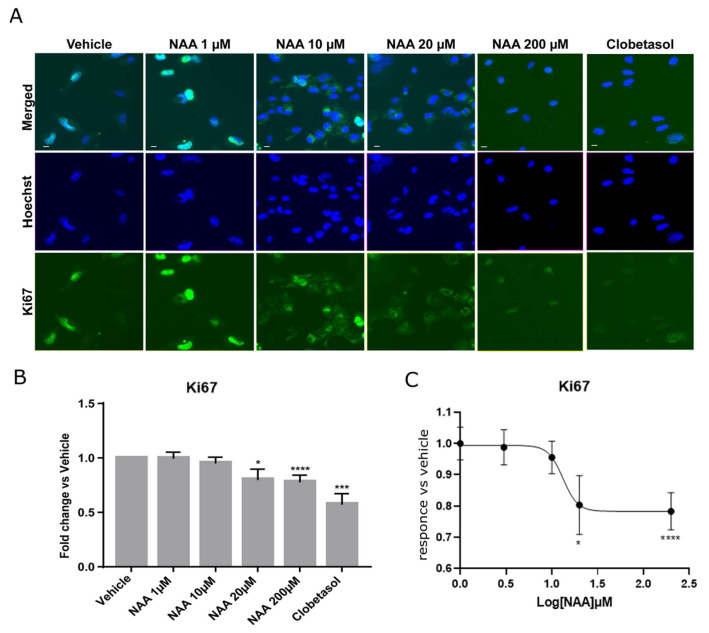
NAA inhibits Oli-neuM proliferation. (**A**) Representative IF images of Oli-neuM cells treated with NAA at the indicated concentrations, with Clobetasol, or with vehicle in DM media for 48 h. Cells were fixed and processed for IF analyses using an anti-Ki67 antibody (green), and nuclei were stained with Hoechst (blue). (**B**) Oli-neuM proliferation was significantly inhibited by 20 μM and 200 μM NAA. Quantification of the Ki67 levels in IF images. Data is the mean intensity of the Ki67 signal (±SEM) in the nucleus normalized to the vehicle control (arbitrarily set to 1). N = 3, n = 75. (**C**) Non-linear regression curve analysis was performed using Graph Pad (software v.9) with the equation “log(inhibitor) vs. response-Variable slope” (Y = Bottom + (Top-Bottom)/(1 + 10^((LogIC50-X)*HillSlope)). * *p* < 0.05, *** *p* < 0.001, **** *p* < 0.0001, two tailed Student’s *t*-test.

The effects of lowering NAA concentrations in the brain as a consequence of disease or injury on the capacity of oligodendroglial precursor cells to differentiate and re-sheath axons have not been explored before. To determine the effects on oligodendroglia proliferation consequent to the lowering NAA concentrations in the brain, we analyzed the levels of the Ki67 protein (Figure 1) at NAA doses ranging from 1 to 200 µM, using immunofluorescence microscopy (IF) followed by multiparametric quantitative image analyses. Ki67 is a nuclear protein that, during active phases of the cell cycle (G1, S, G2, and mitosis), is highly expressed and localizes in the nucleus, while it is poorly detectable and localizes in the cytosol during the quiescent phase (G0). Oli-neuM cells were treated for 48 h in differentiation media supplemented with 1 µM, 10 µM, 20 µM, or 200 μM NAA, 10 µM Clobetasol (used as a positive control for differentiation [30]) or with drug vehicle (DMSO < 0.5%) prior to being processed for IF microscopy. Quantitative image analyses were performed using ScanR 3.0 software (Olympus), as described in Materials and Methods (Figure 1).

We observed that cell proliferation was significantly reduced, starting from NAA concentrations of 20 µM, as indicated by the reduced presence of Ki67 in the nucleus. At 200 µM NAA treatment, proliferation was comparable to that of 10 µM Clobetasol treatment (Figure 1B), which had the lowest proliferation ability among tested samples by promoting differentiation [30]. The analysis of the nonlinear regression curve indicates a bell-shaped response as proliferation reduced between 20 µM and 200 µM, while at 2 mM, it returned to vehicle values. These data suggested that NAA concentrations of 200 µM might be suitable for studying the effects of oligodendrocyte differentiation by NAA treatment.

**Figure 2:**
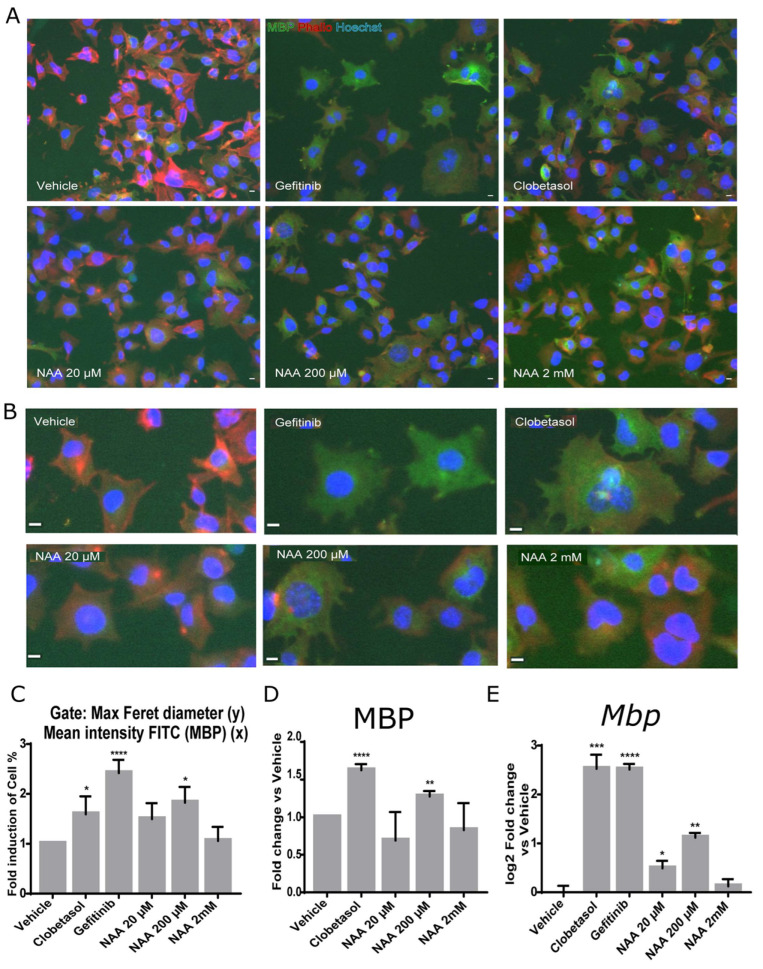
MBP expression and Oli-neuM cell differentiation are stimulated by 200 μM NAA. Oli-neuM cells were plated 24 h in GM and, after media change, treated in DM with the indicated concentration of NAA, 1 μM Gefitinib, 10 μM Clobetasol, or Vehicle (DMSO 0.5% max) for 48 h prior to being processed for (**A**–**D**) quantitative IF image analyses; (**E**) qPCR analyses. (**A**,**B**) Representative IF images are shown for anti-MBP immunostaining (green, FITC). Nuclei were stained with Hoechst (blue) and actin with phalloidin (red, TRITC). Scale bar, 10 μm. (**C**,**D**) IF image quantification data. Quantitative image analysis was performed using (**C**) morphological parameters (Max Feret diameter (y) and Mean Intensity MBP (x)) or (**D**) MBP mean intensity. (**C**) The mean percentage (%) of cells present in the gate (MaxFeretDiameter > 200 (y) and Mean Intensity FITC (MBP) < 100 (x)) normalized vs. vehicle (±SEM; n ≥ 3) was plotted in the graph as fold induction versus vehicle (arbitrarily set to 1). (**D**) Mean intensity of MBP levels in NAA-treated cells. N = 3, n > 1000. (**E**) qPCR analysis of *Mbp* gene expression. Oli-neuM cells were cultured as above and processed for qPCR as indicated in Materials and Methods. Data were normalized vs. vehicle and plotted as log_2_ fold change vs. vehicle ± SEM (n ≥ 3). GraphPad software v.7 was used for graphing and statistical analysis. Two-tailed Student’s *t*-test was used to compare samples to vehicle: * *p* < 0.05, ** *p* < 0.01, *** *p* < 0.001, **** *p* < 0.0001.

To corroborate these data, we analyzed the ability of Oli-neuM cells to differentiate into myelinating OLs using the myelin basic protein (MBP). To follow OL differentiation, we used quantitative IF microscopy, followed by multiparametric image analyses (Figure 2). *Mbp* expression marks the shift from pre-myelinating oligodendrocytes (pre-OLs) to myelinating OLs. Following its transcription, *Mbp* mRNA is transported to endosomal-like structures containing ribonucleic particles, and its translation requires OL maturation and signals derived from neurons [32,33]. It has been suggested that MBP adhesive properties promote the close apposition of the inner membrane leaflets leading to myelin compaction [34]. Because of these characteristics, MBP has been extensively used as a marker to follow OL maturation under drug treatment [20]. Since Oli-neuM cells change cellular morphology while differentiating in mature OLs [29] as do primary oligodendrocytes [33], we gated cells showing enlarged membrane extension and expressing MBP (gate R1 = (MaxFeretDiameter > 200 (y) and Mean Intensity FITC (MBP) < 100 (x)) (Figure 2A,B). The percentage of cells falling into the R1 gate significantly increased after 200 µM NAA treatment (Figure 2C) compared to vehicle. In addition, we determined the levels of MBP in the whole cell (Mean Intensity MBP) after treatment with increasing concentrations of NAA (20 µM, 200 µM, 2 mM NAA, or with 10 µM Clobetasol (Figure 2D). In these experiments, we used 2 mM NAA to get closer to the NAA concentration estimated in immature oligodendrocytes in the brain [1,5,6]**.** In these tests, we used 1 µM Gefitinib or 10 µM Clobetasol treatment as positive controls for promyelinating drugs. We have previously shown that both these drugs can promote *Mbp* gene expression to similar levels [29,30]; while they differ slightly in membrane expansion, Clobetasol differentiated cells have a larger membrane area than Gefitinib (Figure 2B).

Results confirmed that a 200 µM-NAA treatment significantly promotes Oli-neuM differentiation (Figure 2), as shown by the enlargement of the Oli-neuM plasma membrane (Figure 2A–C) and by the increase in MBP levels as estimated by IF microscopy quantitative image analyses (Figure 2D). Importantly, *Mbp* gene expression followed the same pattern (Figure 2E) as determined using qPCR, and the *Mbp* oligos indicated in Table 1. Interestingly, a dose-dependent increase in *Mbp* gene expression was observed from 20 to 200 µM, while 2 mM NAA treatment did not affect either MBP gene expression or membrane enlargement (Figure 2C–E). This result was supported by immunoblot analyses of protein extracts from cells treated with 20 µM or 200 µM NAA (Supplementary Figure S1A).

Altogether, these data support the view that when the extracellular concentration of NAA is lowered from normal conditions (around 10 mM [35]) to a concentration of 200 µM, the surrounding oligodendroglia cells respond by promoting *Mbp* gene expression and cell differentiation.

### 3.2. NAA Treatment Promotes 3-Hydroxy-3-Methylglutaryl-CoA Reductase Increase, Temporally-Regulated Myelin Gene Expression, and Synthetic Axon Myelination

**Figure 3:**
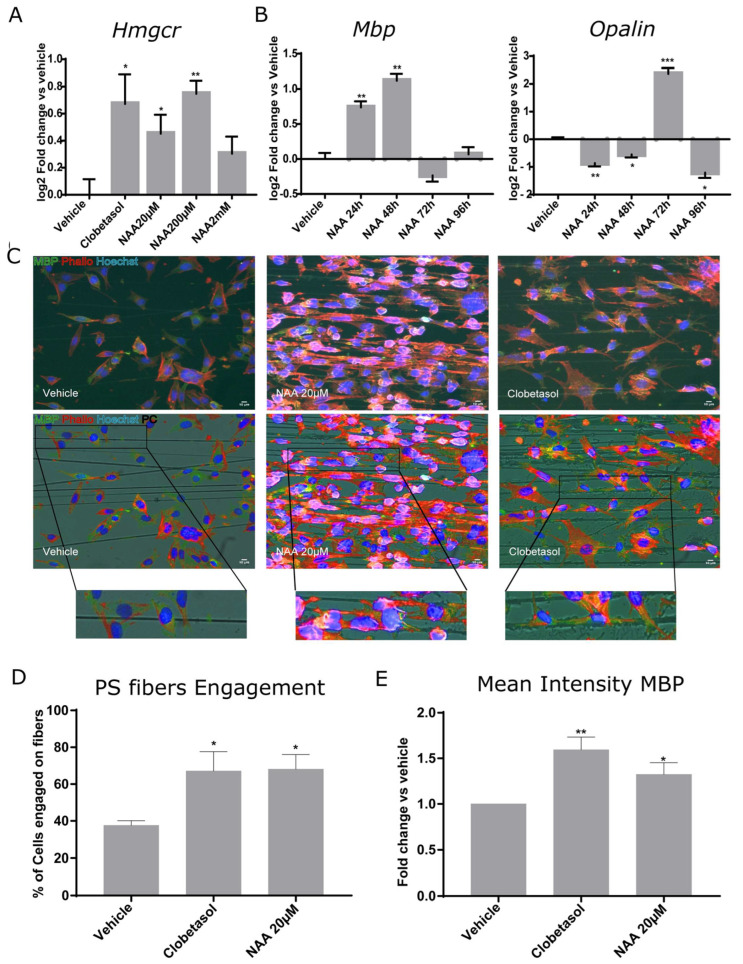
Oli-neuM differentiation and PS fiber engagement are stimulated by NAA treatment. (**A**,**B**) Quantitative RT-PCR analysis of *Hmgcr*, *Mbp,* or *Opalin* gene expression. Oli-neuM were grown in GM 24 h prior to treatment with the indicated NAA concentration, 10 μM Clobetasol, or vehicle (DMSO 0.5% max); (**A**) *Hmgcr* gene expression was determined after 48 h treatment with the indicated compounds. (**B**) *Mbp* and *Opalin* gene expression was determined at the indicated time points. Data were plotted as log_2_ fold change versus vehicle (set at y = 0) ± SEM (n ≥ 3); Statistical analyses were performed using a two-tailed Student’s *t*-test. (**C**,**D**) PS fiber-engagement analyses. (**C**) representative IF images of Oli-neuM cells cultured in chambers containing aligned PS microfibers. MBP (green); Phalloidin (red); Hoechst (nuclei, blue). PC = Phase contrast. Scale bar, 10 μm. Lower panels: Fluorescence and phase contrast merged images. Bottom panels: enlargement of the engaged cell. (**D**) Quantification of fiber engagement. Engagement was estimated as indicated in Materials and Methods and as previously described [20]. Briefly, the mean percentage of engaged cells (±SEM) was estimated on 75 images per sample (n = 3) by counting nuclei located on fibers and having membrane extended along the fiber, as determined by phalloidin (red) staining of actin and MBP (green). (**E**) Quantification of fiber engagement of the mean intensity of MBP in engaged cells. The mean intensity MBP calculated on the engaged cells ± SEM (N ≥ 3) was plotted as fold change vs. vehicle. * *p* < 0.05, ** *p* < 0.01, *** *p* < 0.001, one-tailed paired Student’s *t* test.

Since remyelination is a process that requires novel cholesterol synthesis, and 3-hydroxy-3-methylglutaryl-coenzyme A reductase (HMGCR) is the rate-limiting enzyme in the cholesterol biosynthesis pathway and the target of cholesterol-lowering drugs [36], we determined if NAA concentration could affect *Hmgcr1* gene expression. Consistent with the idea that lowering the extracellular concentration of NAA from 2 mM to 20 µM stimulates lipid synthesis, we observed a significant increase in *Hmgcr1* mRNA at 200 µM NAA (Figure 3A).

As previously mentioned, myelin gene expression is a multi-step process. *Mbp* is among the earliest myelin genes expressed by myelinating OLs, and its expression unlocks the subsequent expression of late genes [37]. Oligodendrocytic myelin paranodal and inner-loop protein (OPALIN) follows MBP expression, being involved in node organization [38]. Following *Opalin* gene expression, OPCs are expected to engage axons. Therefore, we followed the gene expression of *Mbp* and *Opalin* in parallel samples, taken at 24, 48, 72, or 96 h after Oli-neuM treatment with 200 µM NAA in DM. We used 10 µM Clobetasol and vehicle (DMSO 0.5% max) as positive or negative controls, respectively (Figure 3B).

qPCR data indicated that 200 µM NAA treatment strongly stimulated *Mbp* gene expression from 24 h to 48 h after treatment and declined at 72 h (Figure 3B). *Opalin* gene expression strictly followed *Mbp* gene expression, as its mRNA had a sharp peak at 72 h and declined after this time point (Figure 3B, respective panels). This is consistent with the fact that OPALIN is incorporated into engaging fibers and suggests that Oli-neuM treated with NAA can engage axons after 72 h treatment.

To determine if NAA treatment can promote oligodendroglia engagement of synthetic PS axons, we performed engagement tests at 72 h from 20 µM-NAA treatment in DM. Cells were grown in chambers containing aligned electro spun polystyrene (PS) microfibers of 2–4 µm diameter, as previously described [29]. This analysis is currently used widely to test a drug’s ability to promote axon engagement in vitro [28,29,39,40,41]. We chose to start from the minimal concentration at which NAA showed reduced proliferation (Figure 1B,C) and increased *Mbp* gene expression (Figure 2E), namely 20 µM. Cell engagement was measured by counting the percentage (%) of cells whose nucleus was located on the PS fibers and that showed cell membrane wrapping of the fibers (Phalloidin-TRITC labelling of F-actin) and expressing MBP (FITC) elongated onto the polystyrene fibers (Figure 3C). These analyses suggest that lowering NAA to 20 µM stimulates Oli-neuM cells to engage synthetic axons (Figure 3C–E).

### 3.3. Oli-neuM Deacetylation Regulates MBP Gene Expression under NAA Treatment

How NAA can promote OL maturation remains to be established. Previous studies suggest that neuronal-derived NAA signals support or maintain myelination through epigenetic mechanisms in oligodendrocytes [24,31]. Another study suggested that neuronal mitochondrial dysfunction may play a role in compromised myelination by oligodendrocytes in MS by limiting the availability of NAA-derived acetate required for the synthesis of myelin lipids [42].

We, therefore, determined the total protein-acetylation state of Oli-neuM cells treated with NAA from 20 µM to 2 mM in parallel with vehicle and 10 µM Clobetasol by quantitative IF using AKL5C1 anti-AcLys Ab.

**Figure 4:**
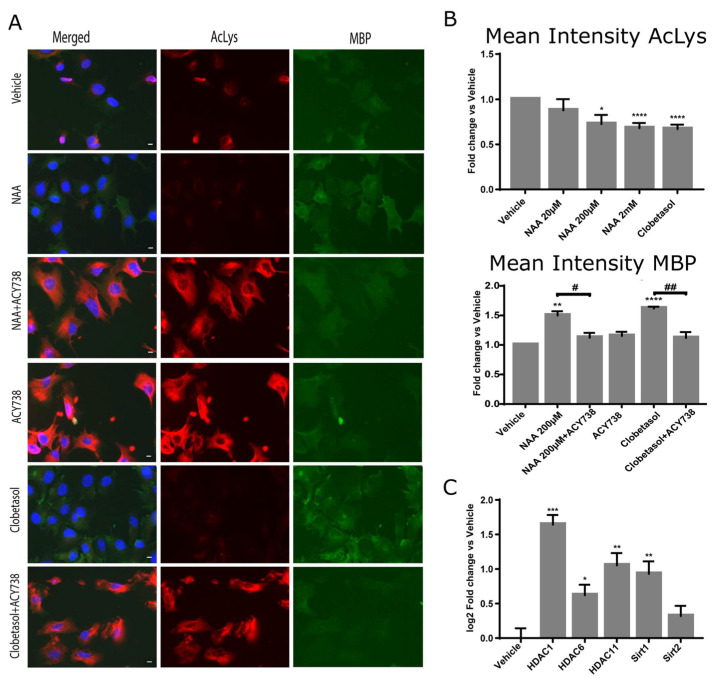
HDAC activity is required for MBP expression under NAA treatment. (**A**,**B**) The total acetylation in Oli-neuM cells treated with indicated NAA dosages was determined using IF analyses. Cells were plated in 96-well plates and treated with the indicated doses of NAA, 10 μM Clobetasol, or vehicle (DMSO 0.5% max) alone or in combination with the HDAC inhibitor ACY-738 in differentiation media for 48 h prior to being processed for IF analyses. (**A**) Representative IF images are shown. Hoechst (blue); anti-AcLys (red); anti-MBP (green). (**B**) Total acetylation was measured using ScanR analyses software (Olympus, v.2.1) tools on at least 75 images per sample of 3 biological replicates. The mean intensity AcLys (TRITC) and MBP (FITC) was calculated and plotted in the graphs as fold change vs. vehicle arbitrarily set to 1 using GraphPad software v.7.0. (**C**) Analyses of HDACs gene expression after NAA treatment. Oli-neuM cells were treated with 200 μM NAA or vehicle control. Changes in gene expression (log_2_ fold change vs. vehicle) were measured by qPCR as indicated in Materials and Methods. Mean values ± SEM. n = 3. * *p* < 0.05, ** *p* < 0.01, *** *p* < 0.001, **** *p* < 0.0001, two-tailed Student’s *t*-test. # < 0.05; ## < 0.01, ANOVA.

We observed that increasing dosages of NAA reduced rather than stimulated protein acetylation. These data support the view that NAA is not a source of acetyl-CoA for acetylation but rather activates HDAC activity. If this is true, then inhibition of HDAC activity should impair MBP expression. We, therefore, estimated the cellular MBP levels by IF quantitative microscopy under treatment with 10 µM of the HDAC inhibitor ACY-738 alone, in combination with 200 µM NAA, or with 10 µM Clobetasol, along with relative controls (Figure 4). In both NAA or Clobetasol and ACY-738 co-treated cells, MBP levels significantly decreased compared to the respective single treatment (Figure 4, Mean intensity MBP panel). These results were confirmed by immunoblot analyses (Supplementary Figure S1B). Altogether, these data show that HDAC activity is necessary for MBP expression. To further corroborate the idea that histone deacetylases are activated upon 200 µM NAA treatment, we determined the levels of expression of the major HDACs involved in cell differentiation in the brain. We focused on HDAC1, HDAC6, HDAC11, SIRT1, and SIRT2, which have been directly related to OL differentiation in humans [43] and mice [31,37,43,44,45,46,47]. We observed that, except for *Sirt2*, all the tested HDACs significantly increased their mRNA after 48 h treatment with 200 µM NAA. *Hdac1* and *Hdac11* gene expression was the most sensitive to treatment (Figure 4C).

We concluded that NAA promotes protein deacetylation and HDAC upregulation and that this activity favours MBP expression during oligodendrocyte differentiation. These data suggest that lowering NAA concentrations below 200 µM in the extracellular space of oligodendrocytes results in a promyelinating stimulus: oligodendrocyte reactivation; differentiation; and novel axon engagement.

## 4. Discussion

NAA fluctuation in the brain has an intimate relationship with neuronal metabolic distress and demyelination. Here, we tested the hypothesis that changes in NAA extracellular levels in the brain, such as those occurring during TBI and SPMS pathologies, by signaling neuronal distress and demyelination, might reactivate oligodendrocyte precursor maturation and stimulate axon engagement. Altogether, our data support this view, as we showed that when NAA dropped from 2 mM to 200 µM, HDACs were activated, proliferation was reduced, and proteins were deacetylated. Importantly, inhibition of HDAC activity in the presence of low NAA levels resulted in the inhibition of MBP expression.

Oli-neuM, by expressing MyRF and GPR17, is representative of premature oligodendrocytes [48]. When stimulated with a promyelinating drug, such as Clobetasol or Gefitinib, this preOL cell line starts to express myelin genes in a regulated manner and, after 72 h from treatment, can engage synthetic axons [28]. Here, we showed that changes in the NAA extracellular concentration strongly affect Oli-neuM differentiation by stimulating gene expression, in a dose-dependent manner, of several genes involved in remyelination. The sequential and temporally-regulated expression of early and late myelin genes, such as *Mbp* and *Opalin*, respectively, is required for proper remyelination of axons in the adult brain [37,38,48]. In this study, we showed that *Mbp* gene expression was stimulated by NAA when its concentration was equal to or below 200 µM within 48 h from treatment, while *Opalin* peaked at 72 h. Furthermore, we observed that between 72 and 96 h from treatment with 20 µM NAA, Oli-neuM appeared to wrap synthetic axons, as established using immunofluorescence microscopy in vitro engagement tests. Since we did not observe Oli-neuM differentiation responses by NAA treatment at a concentration of 2 mM and in normal healthy conditions, NAA levels in oligodendrocytes reached about 10 mM [1,5,6], our data support the view that premyelinating OLs might be reactivated by NAA dropping below 200 µM. We concluded that the optimal concentration of NAA that stimulates resident premyelinating OLs to remyelinate is between 20- and 200 µM NAA. At this concentration, NAA acts as a potent differentiation stimulus for surrounding promyelinating OLs. Our data suggested that NAA can promote PS fiber engagement even at a concentration of 20 µM. This probably reflects the need for cells to remyelinate axons in severe neuronal functional deficit, while OLs must remain quiescent at high NAA dosages. A limitation of this study is the lack of an OPC cellular model for TBI. Moreover, NAA is readily metabolized by OPCs, so further work is necessary to consolidate these data in disease-relevant models. Interestingly, NAA levels within this range were observed in patients after 70 h from TBI [5,7], and reduced NAA concentrations were also found in Secondary Progressive MS patients [8]. It has been shown recently that parenchymal OPCs surrounding demyelinated areas actively contribute to remyelination, but the signal that promotes their reactivation remains unknown [18,48]. NAA, which is abundantly present in the brain and metabolized by oligodendroglia cells, might represent a good candidate to be the sensor molecule for the response to demyelination processes and a potential remyelination drug target.

The idea that a brain metabolite could induce endogenous remyelination cooperating with promyelinating drugs has been previously put forward by metabolomic studies. Global metabolomics approaches have shown that taurine is significantly altered during the course of OPC differentiation, directly impacting cell fate, and supplements of taurine together with promyelinating agents have been shown to improve remyelination in vitro [23]. Our data support the idea that endogenous molecules participate in the signaling that activates the remyelination program, and supplementation with endogenous metabolites, such as NAA, in remyelination therapies could improve promyelinating drug activity in vivo. The idea that OPCs might have an active role as sentinels or as innate immune cells in the CNS by sensing neuron activity via NAA signaling has been recently put forward by others [18,49]. Promyelinating OLs surrounding axons might be considered “sentinels”, which sense normal neuronal activity by capturing NAA concentration levels, thereby discriminating between “normal” vs. “pathological” neuronal activity [48].

From a mechanistic point of view, we showed that NAA promotes OL differentiation by promoting protein deacetylation. This was confirmed by the independent finding that HDACs gene transcription was upregulated. Importantly, we showed that increased MBP under treatment conditions requires histone deacetylation, as the HDAC inhibitor ACY-738 impairs MBP upregulation at a 200 µM NAA concentration. Since ACY-738 reduces MBP expression not only under NAA but also under Clobetasol treatment, it is tempting to speculate that increased HDAC activity is required to promote remyelination not only under NAA treatment but as a general feature of oligodendrocyte maturation. In agreement with our data, it has been reported previously that global changes in gene expression correlate with decreased protein acetylation in the developing corpus callosum [50]. Epigenetic chromatin remodeling, such as histone modification, has been reported to regulate neural precursor cell-fate specification and differentiation [24].

Looking at the specific effects of NAA deacetylase gene expression, we observed that 200 μM NAA treatment promotes *Hdac1* and *Hdac11* gene expression over other deacetylases. A correlation between the activity of these HDACs and oligodendrocyte differentiation was previously observed. High HDAC1 activity in NPCs has been correlated with active Hedgehog signaling (Hh), and loss of HDAC1 activity suppresses Hh-dependent growth [51,52]. More specifically, it has been demonstrated that HDAC1-mediated deacetylation of Glioma-associated factor 2 (Gli2) on Lys 518 increases GLI2 transcriptional activity [51]. Gli2 is an essential transducer of Hh signals responding to Smoothened activation that we have shown is upregulated during remyelination stimulation [28,29,53,54]. We observed that Gli2 is also upregulated by 200 µM NAA treatment of Oli-neuM (Supplementary Figure S2). *Hdac11* is highly expressed in rat brains, and its specific role in myelination is suggested by the correlation between its functional activity and oligodendrocyte-specific gene expression, including MBP and proteolipid proteins [46,55]. Moreover, *Hdac11* transcript levels are high in female patients with MS, suggesting a clinical significance of *Hdac11* gene expression levels and MS pathology [56]. Finally, we cannot exclude that the positive effects of 200 µM NAA on MBP expression could be due to changes in the levels of TCA cycle intermediates as they can regulate gene expression through other epigenetic mechanisms [57]. Such an idea has been previously suggested by the observation that NAA treatment increases the levels of TCA cycle intermediates in a dose-dependent manner [24].

In conclusion, our data demonstrated that lowering the extracellular NAA concentration in the surrounding space of Oli-neuM cells stimulate differentiation and axon engagement and reduce their proliferation. It increases *Hdacs* gene expression, and HDACs activity is required for MBP expression and oligodendroglia differentiation. Given that a sudden lowering of NAA has been reported in human brains during TBI or in SPMS patients, our data suggest that prompt action to avoid the lowering of NAA concentrations might prevent neurodegeneration in the acute phases of these pathologies.

## Figures and Tables

**Table 1:** . PCR primers used in this study Caption.

Gene Name	Forward	Reverse
***Gapdh***	5′-CCAATGTGTCCGTCGTGGATCT-3′	5′-GTTGAAGTCGCAGGAGACAACC-3′
***Hdac1***	5′-ACAGCAATAGGAGGCCAGTT-3′	5′-TCCCTCCTTGCTTTCTCAGG-3′
***Hdac6***	5′-GGAGACAACCCAGTACATGAATGAA- 3′	5′-CGGAGGACACGGAGGACAGAGCCTGTAG-3′
***Hdac11***	5′-GGGGGATCTCAGTGATGGTA-3′	5′-AAGAGAAGCTGCTGTCCGAT-3′
***Hmgcr1***	5′-CTTGTGGAATGCCTTGTGATTG-3′	5′-AGCCGAAGCAGCACATGAT-3′
***Mbp***	5′-TACCCTGGCTAAAGCAGAGC-3′	5′-GAGGTGGTGTTCGAGGTGTC-3′
***Opalin***	5′-CAGCTGCCTCTCACTCAACATC-3′	5′-TCCCAAAGGCAGACTTCTCTCG-3′
***Sirt1***	5′-GTAAGCGGCTTGAGGG-3′	5′-TTCGGGCCTCTCCGTA-3′
***Sirt2***	5′-ATCCACCGGCCTCTATGACAA-3′	5′-CGCATGAAGTAGTGACAGATGG-3′

## Data Availability

Not applicable.

## Supplementary Materials

The following supporting information can be downloaded at: https://www.mdpi.com/article/10.3390/cells12141861/s1, Figure S1. Western blot analyses following treatment with (A) NAA 20 or 200 µM or Clobetasol 10 µM or Gefitinib 1 µM like positive controls or vehicle alone (DMSO < 0.5%) (B) NAA 200 µM, Clobetasol 10 µM or ACY-738 10 µM, alone or in double treatment with ACY-738. Figure S2. 200 µM NAA treatment promotes Gli2 gene expression in Oli-neuM.
