# Supplementary material for: N-Acetylaspartate Drives Oligodendroglial Differentiation via Histone Deacetylase Activation
Source: Cells. 2023 Jul 14;12(14):1861. doi: 10.3390/cells12141861 (PMC10378218; doi:10.3390/cells12141861)
Supplement: Supplementary file 1 [file cells-12-01861-s001.zip › cells-2437470-supplementary.pdf]

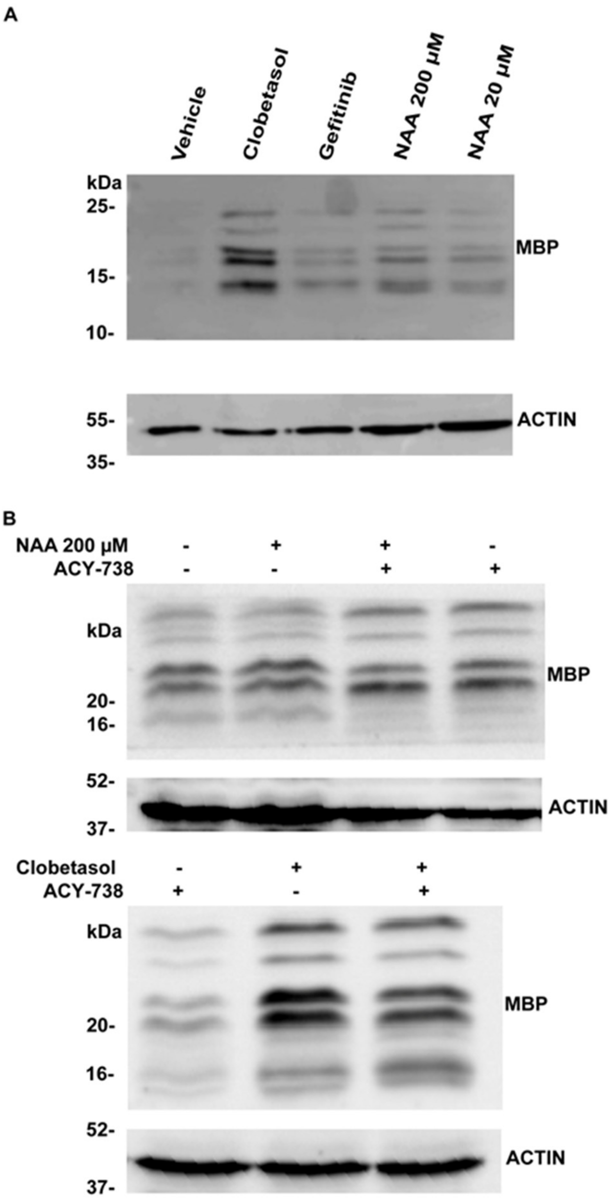

Supplementary Figure S1. Western blot analyses following treatment with A) NAA 20 or 200 μM or Clobetasol 10 μM or Gefitinib 1 μM like positive controls or vehicle alone (DMSO <0.5%) B) NAA 200 μM, Clobetasol 10 μM or ACY-738 10 μM, alone or in double treatment with ACY-738. Treatments are indicated above the blots. The name of protein detected is indicated to the left of each blot, and the molecular weight indicated by the marker is indicated to the right. All gels and blots were run under the same experimental conditions. For sample preparation typically, 2.75 × 10[5] Oli-neuM cells were seeded in 6-well plates in GM media and cells were grown to 70% confluence. Treatments were performed for 48h before protein extraction and the western blotting procedure. The western and immunoblotting was performed as indicated in Materials and Methods.

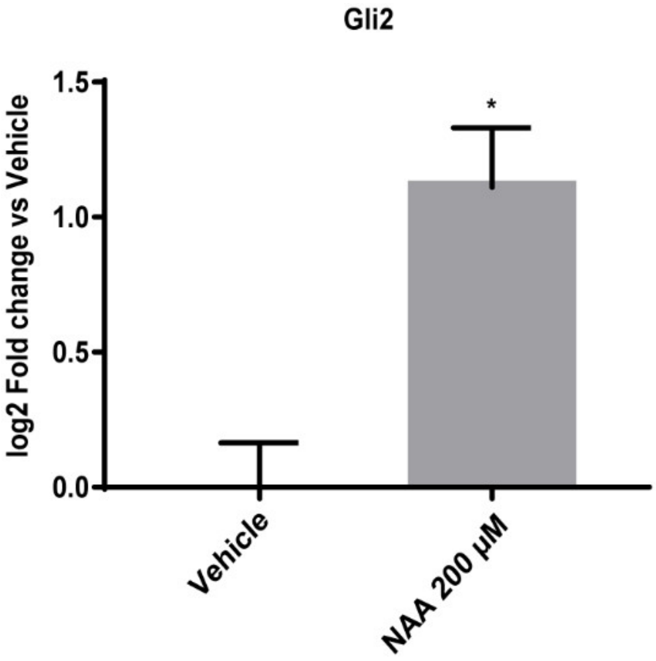

Supplementary Figure S2. 200µM NAA treatment promotes Gli2 gene expression in Oli-neuM. Cells were plated for 24h in GW media prior to media change with DM + 200 µM NAA treatment was proceeded for 48 h prior to mRNA extraction and RT-qPCR analyses. The mean of $\log_2$ fold change was calculated and plotted in the graphs as *$\log_2$ fold change vs vehicle* (±SEM; n=3). GraphPad software was used for graphing and statistical analysis performed with two tail t-student test: *$p<0.05$.
